# Gold Nanorod–Radiopharmaceutical Conjugates for Nuclear Medicine Theranostics: A Methodological and Multiscale Perspective

**DOI:** 10.3390/ijms27104514

**Published:** 2026-05-18

**Authors:** Ludovica Binelli, Andrea Attili, Iole Venditti, Chiara Battocchio, Valentina Dini, Maria Lucia Calcagni, Marco Ranaldi, Giovanna Iucci, Luca Tortora, Sveva Grande, Alessandra Palma, Barbara De Berardis, Maria Grazia Ammendolia, Teresa Scotognella, Francesca Campanaro, Monica Dettin, Lucrezia Bianchi, Antonella Rosi, Andrea Fabbri

**Affiliations:** 1 Sciences Department, Roma Tre University, 00146 Rome, Italy; ludovica.binelli@uniroma3.it (L.B.); iole.venditti@uniroma3.it (I.V.); chiara.battocchio@uniroma3.it (C.B.); marco.ranaldi@uniroma3.it (M.R.); giovanna.iucci@uniroma3.it (G.I.); luca.tortora@uniroma3.it (L.T.); 2 Istituto Nazionale di Fisica Nucleare (INFN), Sezione di Roma3, 00146 Rome, Italy; andrea.attili@roma3.infn.it (A.A.); marialucia.calcagni@policlinicogemelli.it (M.L.C.); teresa.scotognella@policlinicogemelli.it (T.S.); lucrezia.bianchi@uniroma3.it (L.B.); andrea.fabbri@infn.roma3.it (A.F.); 3 National Center of Artificial Intelligence and Innovative Technologies for Health, Istituto Superiore di Sanità, 00161 Rome, Italy; sveva.grande@iss.it (S.G.); alessandra.palma@iss.it (A.P.); barbara.deberardis@iss.it (B.D.B.); maria.ammendolia@iss.it (M.G.A.); 4 Istituto Nazionale di Fisica Nucleare (INFN), Sezione di Roma1, 00185 Rome, Italy; 5 Nuclear Medicine Unit, Fondazione Policlinico Universitario A. Gemelli IRCCS, 00168 Rome, Italy; fra.campanaro99@gmail.com; 6 Department of Radiological and Haematological Sciences, Università Cattolica del Sacro Cuore, 00168 Rome, Italy; 7 Department of Industrial Engineering, University of Padova, 35131 Padova, Italy; monica.dettin@unipd.it; 8 Mathematics and Physics Department, Roma Tre University, 00146 Rome, Italy; 9 Independent Researcher, 00161 Rome, Italy; rosi.antonella58@outlook.com

**Keywords:** gold nanoparticles, drug delivery system, theranostic, Auger electrons, radiobiology, Monte Carlo simulations

## Abstract

The creation of innovative systems that are able to combine diagnosis and therapy is a crucial opportunity in nuclear medicine. Here, we propose a methodological and multiscale approach for the development of a theranostic platform based on AuNRs functionalized with radiopharmaceuticals. AuNRs offer a versatile and effective system due to their unique physicochemical properties and the possibility of surface functionalization with targeting molecules. Within this framework, key challenges include the functionalization of AuNRs to target the cell nucleus, the loading of AuNRs with radiopharmaceuticals, and the investigation of Auger electron emission from AuNRs under gamma irradiation. Multiscale modelling is employed to describe the behaviour of the system within the cellular environment and to predict potential radiobiological enhancement effects, including synergistic interactions between functionalized AuNRs and radiopharmaceutical agents such as ^99m^Tc-sestaMIBI. The experimental activity includes gamma irradiation studies, along with the structural and physical characterization of nanomaterials and in vitro biological investigations on T98G cells, to evaluate cytotoxicity and metabolic alterations, with the aim of assessing the potential synergistic effects of the combined system.

## 1. Introduction

In the past decade, nuclear medicine has made great progress in cancer diagnosis and treatment through the employment of new radiopharmaceuticals that preferentially target the tumour while preserving healthy tissues [1]. Moreover, there has been an increasing integration between nuclear medicine and nanomedicine, which exploits nanoparticles (NPs), for diagnostic and therapeutic purposes. Among them, gold nanoparticles (AuNPs) are nanometre-sized particles (1–100 nm) with different shapes, such as spheres, rods, stars, and cubes. The main potential advantages of AuNPs are their high surface area/volume ratio, versatile synthesis, high biocompatibility, and easy functionalization. In addition, AuNPs have significant potential for therapeutic applications due to the effective radio enhancement repeatedly demonstrated for irradiation with photon beams [2,3,4,5]. This effect was predicted, at least partially, by theoretical models in which it was suggested that the enhancement in the radiobiological effect could be explained by the strong difference in energy absorption between high-Z AuNPs and water, resulting in an increase in the local dose deposition in cells [6,7,8,9,10,11]. However, several studies challenge this purely physical mechanism hypothesis and affirm the importance of oxidative stress in this process [5,12,13]. At present, despite the increasing amount of data regarding AuNP-induced radiosensitization, it is still difficult to draw conclusions regarding the effect due to the diversity of parameters and conditions (nanoparticle size, cell lines, radiation source, administration route, etc.) used in the literature, and there are no definitive answers about the responsible mechanism [14,15]. A better understanding through further aimed studies remains an essential step towards the clinical use of AuNP radiosensitizers.

In recent years, gold nanorods (AuNRs) have attracted increasing interest in the biomedical field, thanks to their peculiar chemical–physical properties, such as the presence of a double plasmon peak, due to the Localized Surface Plasmon Resonance (LSPR) effect, and the possibility of differentiated functionalization [16,17,18,19,20]. Thanks to their specific properties, AuNRs can offer a significant contribution to nuclear medicine, in the field of the delivery of radiopharmaceuticals to diagnose, treat, and follow diseases, including cancer [21,22,23,24,25].

To date, despite the growing interest in AuNRs for biomedical applications, the development and systematic investigation of hybrid systems combining AuNRs with clinically relevant radiopharmaceuticals—particularly those designed for nuclear targeting—remain extremely limited. Studies involving ^99m^Tc-labelled AuNRs are scarce and largely lack a mechanistic and quantitative evaluation of their potential synergistic effects [26].

^99m^Tc is known to emit 140 keV gamma rays and to have a half-life of 6 h, and it can be easily eluted and handled using a high-specific-activity ^99^Mo/^99m^Tc generator; for these reasons, ^99m^Tc is used for diagnostic purposes. Additionally, ^99m^Tc emits less than 1% Auger electrons upon decay compared to 3.7–19.9% for iodine-125 (125I), iodine-123 (123I), and thallium-201 (201Tl). However, some potential advantages have been highlighted, including: a short half-life, Auger electron energies ranging from 0.9 to 15.4 keV, and good availability. In addition, its imaging-friendly characteristics can enable therapy monitoring and follow-up. The possibility of combining AuNRs with radiopharmaceuticals therefore opens a strategic avenue toward hybrid systems capable of exploiting both the molecular specificity of radiopharmaceuticals and the physical advantages of nanomaterials.

**Figure 1:**
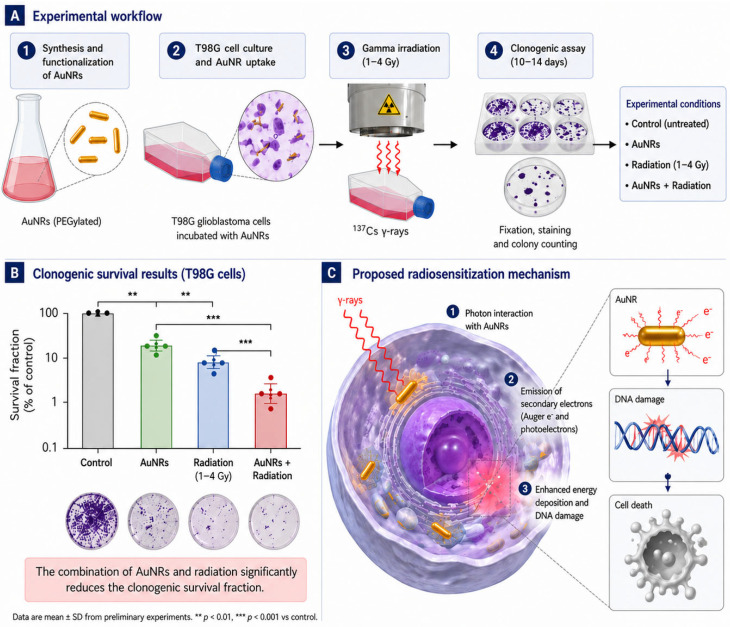
Experimental workflow, preliminary clonogenic survival, and proposed mechanism of AuNR-mediated radiosensitization in T98G cells. (**A**) Experimental workflow including AuNR synthesis and functionalization, incubation with T98G cells, gamma irradiation (1–4 Gy), and clonogenic assay. (**B**) Representative clonogenic survival showing reduced survival after AuNRs or radiation alone and further decrease after combined treatment. (**C**) Proposed mechanism of radiosensitization based on enhanced local energy deposition from secondary electrons emitted by AuNRs upon irradiation, leading to increased DNA damage and reduced cell survival.

Preliminary studies in T98G glioblastoma cells, performed to evaluate the biological response to AuNR exposure, gamma irradiation (1–4 Gy), and their combination, have shown that treatment with AuNRs alone or irradiation alone resulted in a moderate reduction in survival fraction (SF) as measured by cell killing, consistent with previously reported data. Notably, the combined treatment (AuNRs + gamma irradiation) induced a significantly enhanced decrease in cell survival compared to either condition alone, suggesting a potential radiosensitizing effect of AuNRs (see Figure 1) [23].

Although these results are preliminary, they support the hypothesis of a synergistic interaction between nanostructures and ionizing radiation. Understanding the type and extent of this radionuclide–AuNR interaction through modelling studies and biological experiments (including metabolic profiling and ultrastructural investigation) is the main goal of SEGNAR (Synergic Effects of Gold Nanorods And Radiopharmaceutical), a three-year Istituto Nazionale di Fisica Nucleare (INFN)-funded project, which addresses the development of a hybrid AuNR-based platform functionalized for nuclear targeting and combined with clinically relevant radiopharmaceuticals, with the hypothesis that the nanoscale localization of Auger electron emitters may enhance radiobiological effectiveness through synergistic mechanisms.

## 2. Multidisciplinary Approach and Scientific Rationale

The development of theranostic platforms based on functionalized AuNRs requires the integration of complementary expertise spanning radiation physics, nanochemistry, radiobiology, and nuclear medicine. These hybrid nanosystems represent multifunctional agents in which diagnostic imaging and therapeutic activity are intrinsically combined, enabling the quantitative evaluation of nanoparticle biodistribution together with localized radiation dose amplification at the cellular level.

From a radiation physics perspective, a key challenge concerns the quantitative description of the interaction between ionizing radiation and high-Z nanostructures, particularly with respect to Auger electron cascades and the resulting nanoscale energy deposition patterns. While macroscopic dose enhancement effects are relatively well characterized, predicting energy deposition at the subcellular scale remains difficult due to the strong dependence on nanoparticle localization and intracellular microenvironment. Monte Carlo and track structure simulations are therefore essential to support the interpretation of experimental observations and to establish correlations between localized physical dose enhancement and biological endpoints.

In parallel, the chemical engineering of AuNR-based nanoplatforms plays a critical role in determining theranostic performance. Controlled synthesis and reproducible surface functionalization are required to ensure colloidal stability, biocompatibility, and efficient intracellular delivery. In particular, surface modification strategies enabling selective subcellular targeting are highly relevant for maximizing the therapeutic effectiveness of short-range Auger electron emitters. Within this framework, the incorporation of clinically established radiopharmaceuticals represents a promising strategy to facilitate translational applicability. Among potential candidates, technetium-99m sestaMIBI (^99m^Tc-sestaMIBI) is particularly attractive due to its established clinical use as an imaging agent and its potential contribution as an intracellular Auger electron source, although its effective role in nanoscale dose amplification remains to be fully clarified.

Radiobiological investigations are essential to determine whether increased nanoscale energy deposition translates into enhanced biological effectiveness. Quantitative in vitro studies addressing cytotoxicity, DNA damage induction, and metabolic alterations are therefore necessary to identify the relative contribution of physical dose enhancement and biological response modulation, as well as possible synergistic interactions between nanoparticle-mediated radiosensitization and radionuclide emission.

From a nuclear medicine perspective, the integration of imaging capability within the same nanoplatform enables the quantitative evaluation of nanoparticle distribution and supports the development of image-guided treatment strategies. The use of clinically validated radiopharmaceuticals facilitates compatibility with existing diagnostic workflows and enables the implementation of quantitative Single-Photon Emission Computed Tomography (SPECT)-based dosimetry approaches. However, several aspects remain to be addressed, including radionuclide–nanoparticle co-localization efficiency, intracellular retention kinetics, and the predictive value of imaging-derived dosimetric parameters for therapeutic response.

**Figure 2:**
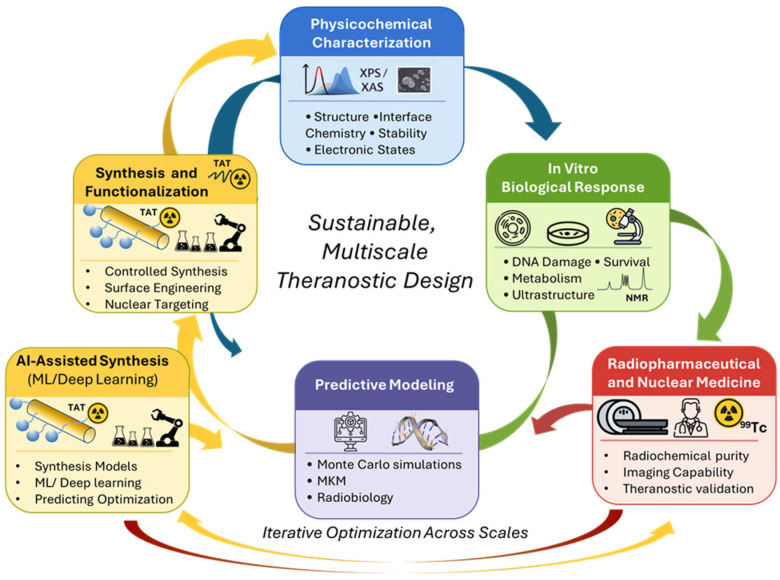
A schematic representation of the multidisciplinary workflow underlying the development of the AuNR-based theranostic platform, illustrating the integration of modelling, nanomaterial synthesis, radiopharmaceutical functionalization, and biological validation steps and highlighting their interconnections.

Overall, AuNR-based theranostic platforms represent a promising strategy for integrating quantitative imaging with localized radiation dose amplification at the nanoscale. Further advances in radiation transport modelling, nanoparticle surface engineering, radiopharmaceutical functionalization, and mechanistic radiobiological validation will be essential to support the development of clinically relevant Auger-based nanotheranostic approaches (see Figure 2).

### 2.1. Predictive Modelling

The modelling of the AuNR-based system in the cellular environment focuses on predicting radiobiological enhancement following irradiation, as well as identifying potential synergistic mechanisms between AuNR–TAT and the radiopharmaceutical ^99m^Tc-sestaMIBI.

Previous modelling approaches for AuNP systems devised to explain the observed radiobiological enhancement are mainly based on the Local Effect Model (LEM) [7,9,10]. These approaches describe the enhancement primarily in terms of the direct effect of the local dose deposited by secondary electrons produced in the AuNPs (Auger and photoelectrons). However, they do not explicitly account for the indirect radiation effects mediated by the chemical environment, which may be significantly affected by the presence of AuNPs. Therefore, such models explain the enhancement only through short-range effects assuming the localization of the AuNPs inside the cell nucleus, a condition that is not always verified in the experiments where the enhancement has been observed [11,15].

These limitations can be addressed by integrating nanodosimetric simulations with Geant4-DNA [27,28,29,30,31,32] (a Geant4 extension) and a Microdosimetric Kinetic Model (MKM) [33,34]. The use of Geant4-DNA enables the accurate characterization of secondary electron spectra and energy deposition at the micro- and nanometric scales by tracking low-energy electrons. The statistical reliability of Monte Carlo results is ensured by simulating a sufficiently large number of particle histories to achieve the convergence of the calculated physical quantities, with associated uncertainties estimated from the variance in the simulated distributions. Of relevance are electrons generated by the interaction of primary radiation (external photon beams or internal ^99m^Tc sources) with biological tissues and AuNR-based systems (AuNRs, AuNR–TAT, and AuNR–TAT–^99m^Tc-sestaMIBI).

Although the original formulation of the MKM does not explicitly include indirect radiation effects, recent extensions of the model couple DNA repair kinetics with radiochemical processes affected by the cellular chemical environment (see, for example, [35]). Within these recent frameworks, the MKM can be applied to model AuNR–^99m^Tc-based or similar radiopharmaceutical systems. This enables the evaluation of biological effects following the irradiation of T98G cells with and without AuNRs (including contributions from primary particles and secondary electrons generated in both tissue and AuNR systems) in terms of predicted lesion yield and cell survival probability. It should be noted that the proposed mechanisms remain partially hypothetical and require experimental validation. Alternative contributions, including indirect radiation effects and biologically mediated responses, are also likely to play a role.

### 2.2. Synthesis, Functionalization and Characterization of AuNRs

The synthesis of engineered and functionalized AuNRs is progressively evolving toward increasingly complex systems, ultimately aiming at the delivery of a radiopharmaceutical coupled to AuNRs into the cell nucleus. The goal is to have a set of well-defined models that allow for highlighting and identifying the multiple chemical and physical mechanisms that combine synergistically. Drug Delivery Systems (DDSs) based on gold nanoparticles have been successfully developed; however, their application to the targeted delivery of a radiopharmaceutical to the nucleus of tumour cells remains largely unexplored [17,36].

Current synthesis strategies focus on the functionalization of AuNRs with Cetyltrimethylammonium Bromide (CTAB) to achieve high yield, low polydispersity, and adequate stability in cell culture media. Established two-step synthetic approaches have been reported to provide reproducible AuNRs with controlled physicochemical properties [37,38]. Building on these methodologies, AuNRs can be further functionalized with a TAT peptide containing a free cysteine to anchor it to the gold surface, promoting nuclear localization. TAT, derived from HIV-1, was identified in 1988 as the first member of the class of cell-penetrating peptides (CPPs). CPPs are defined as short peptide sequences, typically comprising up to 30 amino acids, which are capable of crossing cell membranes and delivering bioactive cargo into cells. Their membrane translocation ability is commonly attributed to the presence of positively charged amino acids, such as arginine and lysine, at physiological pH.

The amino acid sequence of the TAT peptide is as follows:

H–Gly–Arg–Lys–Lys–Arg–Arg–Gln–Arg–Arg–Arg–Pro–Gln–NH_2_

The use of TAT peptides has found broad application in biomedical research and therapy, owing to their ability to transport chemotherapeutic agents, nucleic acids, and gold nanoparticles across cellular membranes. In this context, the incorporation of ^99m^Tc-sestaMIBI into AuNR–TAT systems represents a promising strategy, maintaining the compromise between the loading efficiency and colloidal stability of the engineered system.

The morphology and molecular structure stability of AuNR-based nanosystems can be investigated using a combination of complementary spectroscopic techniques, including FT-IR, UV–vis, XPS, and Synchrotron Radiation (SR)-based methods such as SR-X-ray Photoelectron Spectroscopy (XPS), Near-Edge X-ray Absorption Fine Structure (NEXAFS) and X-ray Absorption Spectroscopy (XAS), both before and after conjugation with ^99m^Tc-sestaMIBI. The radiopharmaceutical itself has been extensively characterized by the same techniques, enabling an accurate description of the molecular, electronic, and local structure around the technetium centre [39,40,41,42].

Such a multitechnique approach allows for the identification of spectral changes that may be associated with chemical interactions arising at the ^99m^Tc-sestaMIBI/nanoparticle interface [23,43]. In particular, spectroscopic analysis provides critical insight into the structural stability of AuNRs upon functionalization with TAT and subsequent interaction with ^99m^Tc-sestaMIBI, as well as into the nature of the functional groups of TAT and ^99m^Tc-sestaMIBI involved in surface binding. Special attention is warranted for elucidating the nature of the AuNR–TAT interaction, distinguishing between chemisorption (stable covalent bonding) and physisorption (non-covalent intermolecular interactions).

With respect to the ^99m^Tc-sestaMIBI/AuNRs interface, achieving a detailed understanding of its electronic and local structure is expected to represent a key element for interpreting radiation-induced processes and for informing the development of more realistic radiobiological models.

The colloidal stability of AuNRs, AuNR–TAT, and AuNR–TAT–^99m^Tc-sestaMIBI in biological fluids and cell culture media represents a key aspect in the development of the nanoscale radiotherapeutic system to understand the complex interactions with the biological environment. Nanoparticle stability critically influences biological activity, cellular uptake, biodistribution, and safety, thereby shaping AuNR–cell interactions and overall therapeutic potential. In serum-containing media or upon entry into the bloodstream, AuNRs undergo complex interactions with proteins, ions, and small biomolecules that can modify surface chemistry, reduce electrostatic repulsion, and enhance van der Waals attractions, promoting agglomeration [44]. Such effects have also been reported in the presence of thiol-containing molecules, including glutathione and cysteine, as well as under high-osmolarity conditions [45,46,47]. Protein adsorption and protein corona formation may further exacerbate agglomeration through protein–protein interactions [48], with profound consequences for tumour targeting, intracellular trafficking, nuclear localization, and pharmacokinetics and potential adverse effects such as vascular obstruction [45]. Temporal stability is commonly evaluated through measurements of hydrodynamic diameter and ζ-potential, typically using Dynamic Light Scattering (DLS) and electrophoretic mobility analysis [49]. Both short- and long-term stability profiles provide insight into aggregation kinetics under physiologically relevant temperature conditions.

### 2.3. In Vitro Biological Studies

Biological investigations in glioblastoma models could be conducted using T98G cells, a radioresistant cell line grown as monolayers and exposed to AuNRs, AuNR–TAT, and AuNR–TAT–^99m^Tc-sestaMIBI, in both the presence and absence of gamma irradiation within clinically relevant dose ranges. Such in vitro models provide a suitable framework for exploring multiple biological endpoints, including clonogenic survival, DNA damage, metabolic alterations, and ultrastructural changes. Moreover, experiment designs should include appropriate controls, including untreated cells, irradiation-only conditions, and nanoparticle-only treatments, to enable a robust assessment of potential synergistic effects. The biological investigations will be conducted in at least biological independent triplicate, with experimental conditions, including radiation doses (e.g., 1–4 Gy) and nanoparticle concentrations, that are selected based on previously published studies [23] and will be further optimized depending on the specific experimental endpoint.

Cytotoxic effects are commonly assessed through clonogenic assays, which remain the gold standard for evaluating radiation-induced cell killing and long-term proliferative capacity [50]. In parallel, cellular metabolic status can be monitored using colorimetric assays such as MTT, enabling comparison under conditions consistent with clonogenic experiments. DNA damage is typically evaluated using complementary fluorescence-based approaches that detect double-strand breaks (DSBs) and chromosomal damage. The use of immunofluorescence to track γ-H2AX foci provides critical insights into the kinetics of DSB induction and repair, while micronucleus assays offer a definitive measure of irreversible chromosomal fragmentation [51,52]. Dose–response relationships will be systematically investigated, as they are essential for deriving radiobiological parameters and validating predictive models.

Integrating automated image analysis further ensures that these complex biological interactions are quantified with the high throughput and reproducibility required for translating nanomedicine from the bench to prospective clinical application.

Beyond cytotoxicity and genotoxicity, alterations in cellular metabolism represent a critical dimension of the biological response to combined nanomaterial and radiation exposure. Magnetic Resonance Spectroscopy (MRS) offers a powerful, non-destructive approach for investigating treatment-induced metabolic reprogramming. The metabolic profile of T98G cells has been previously characterized by 1H MRS [53,54], revealing intense levels of reduced glutathione, a hallmark associated with intrinsic radioresistance [55,56]. Moreover, MRS studies on glioblastoma and glioblastoma stem-like cells have highlighted the central role of lipid and energy metabolism in therapy response [57,58]. Accordingly, pathways related to lipid metabolism, glutathione homeostasis, and lactate extrusion emerge as relevant indicators of treatment-induced stress and radiosensitization.

Quantitative endpoints will be normalized to appropriate controls depending on the assay, including untreated and single-treatment conditions, following established protocols reported in the literature [23,53,54]. Specifically, clonogenic survival data will be normalized to the plating efficiency of untreated control cells, while metabolic assay results (e.g., MTT, ^1^H MRS) will be expressed as a percentage relative to untreated controls. DNA damage endpoints (e.g., γ-H2AX foci) will be quantified per nuclei and normalized to control conditions to allow for comparison across treatments. For all biological endpoints, significance will be determined using an ANOVA or Student’s *t*-tests where appropriate, after the verification of normality and homogeneity of variance. When multiple comparisons are performed, appropriate post hoc tests (e.g., Tukey’s or Bonferroni correction) will be applied. Variability will be expressed as the Standard Error of the Mean (SEM), in line with standard practice in radiobiology.

Transmission Electron Microscopy (TEM) represents a key technique for visualizing AuNR–cell interactions at the ultrastructural level. Owing to their electron-dense nature, AuNRs can be directly localized within cellular compartments, enabling the analysis of uptake pathways, intracellular trafficking, and subcellular distribution. Numerous studies have demonstrated that AuNR internalization depends on particle geometry, surface chemistry, and cell type [59,60,61,62,63,64]. Cationic surface functionalization often enhances uptake efficiency, while different endocytic mechanisms may coexist within established cell lines. Beyond localization, TEM-based ultrastructural analysis provides valuable information on cellular damage and modes of cell death induced by AuNR formulations alone or in combination with irradiation. Radiation-induced cell death is classically categorized into apoptosis, autophagic cell death, and necrosis, each characterized by distinct morphological hallmarks [65,66,67]. Apoptotic cells exhibit chromatin condensation, cytoplasmic shrinkage, and the formation of apoptotic bodies, whereas autophagic cell death is associated with an accumulation of double- or multi-membraned autophagosomes. Necrosis, by contrast, involves rapid plasma membrane permeabilization, organelle swelling, and the release of intracellular contents, potentially triggering inflammatory responses. In this context, the ultrastructural identification of cell death modalities following treatment with AuNR-based systems, particularly those functionalized with TAT and radiopharmaceuticals and combined with irradiation, may provide mechanistic insight into treatment efficacy and safety. Evidence of specific or mixed death pathways, with or without inflammatory features, may help formulate hypotheses on potential therapeutic outcomes and translational relevance of AuNR-based nuclear-targeted theranostic strategies. Attention should be given to potential confounding factors, including AuNR aggregation, heterogeneity in cellular uptake, and variability in intracellular localization, which may significantly influence the observed biological outcomes.

In addition, biological responses deviating from expected ranges reported in the literature would require further mechanistic investigation to distinguish between experimental variability and biologically relevant treatment-related effects.

These factors collectively contribute to a multi-parametric interpretation of the biological response rather than a single-mechanism explanation.

### 2.4. Radiopharmaceutical and Nuclear Medicine

Radiopharmaceutical-related activities represent a pivotal component in the development of AuNR-based theranostic systems and benefit from a dual approach that integrates both non-radioactive (“cold”) and radioactive (“hot”) formulations. The use of cold analogues, obtained through the decay of the corresponding radioactive compounds, provides a practical and transferable strategy for optimizing loading and handling protocols under conditions that closely mirror those required for radiolabelled systems. SestaMIBI serves as a well-established model radiopharmaceutical, enabling the systematic investigation of conjugation efficiency, stability, and functional performance prior to the transition to ^99m^Tc-labeled formulations. Ensuring high radiochemical purity remains a critical prerequisite for reliable biological and theranostic evaluation, as it directly influences the reproducibility and interpretability of downstream experiments. Once optimized, AuNR–TAT–^99m^Tc-sestaMIBI conjugates offer a unique platform for probing the integration of diagnostic and therapeutic functions within a single nanosystem. Experimental validation in a nuclear medicine setting is essential to assess feasibility across different operational configurations and to support translation from controlled laboratory conditions to clinically relevant environments.

## 3. Expected Impact and Clinical Translation Considerations

AuNR–radiopharmaceutical hybrid systems address key unmet needs in nuclear medicine and oncology by combining improved tumour selectivity, integrated diagnostic–therapeutic functionality, and enhanced biological effectiveness through nanoscale dose amplification. Leveraging the tunable physicochemical properties of AuNRs, these platforms offer a rational strategy to concentrate radiation effects at the subcellular level, potentially overcoming intrinsic radioresistance while limiting off-target toxicity. From a translational standpoint, such systems may support more personalized and adaptive treatment paradigms, including image-guided therapy and combined-modality approaches. However, clinical implementation depends on the ability to ensure reproducible synthesis, scalable and standardized manufacturing, long-term stability, and robust safety profiles. In parallel, regulatory challenges associated with multifunctional nanomedicines must be addressed early, integrating quality by design and regulatory-aware development strategies. Within this context, sustainability represents a unifying principle, spanning environmentally responsible production, clinical feasibility, and long-term translational viability. As envisioned in the SEGNAR framework, the development of AuNR-based theranostic systems aims to align technological innovation with realistic clinical adoption, setting the stage for future advances discussed in the next section.

## 4. Conclusions

The ultra-selective irradiation of nuclear DNA mediated by Auger electron-emitting nanosubstrates may represent a promising strategy to induce localized and potentially more effective radiobiological damage, overcoming resistance to conventional chemo-radiation therapies, although this remains to be validated experimentally. By confining energy deposition to the immediate vicinity of chromatin, this approach may maximize radiobiological effectiveness while limiting off-target effects.

Within this framework, SEGNAR represents an integrative and forward-looking paradigm, combining technetium-based radiopharmaceuticals with the radiosensitizing properties of gold nanostructures into a single multifunctional platform. By assembling established components into a novel configuration, this strategy could potentially contribute to addressing key limitations in current nuclear medicine approaches.

The convergence of nuclear targeting, nanoscale dose modulation, and theranostic functionality may offer new opportunities for more precise and image-guided cancer treatment strategies, particularly in radioresistant tumours. At the same time, attention to reproducibility, scalability, and sustainability is essential to assess the translational relevance of the proposed approach.

Despite its promising perspective, the proposed approach requires systematic experimental validation and extension to multiple biological models to assess its robustness and generalizability.

Ultimately, nuclear-directed nanotheranostics may contribute to a shift in radiation delivery paradigms moving from macroscopic dose escalation toward molecularly precise, biologically optimized intervention, within a hypothesis-driven strategy supported by the existing literature and preliminary observations.

## Data Availability

No new data were created or analyzed in this study. Data sharing is not applicable to this article.

## Abbreviations

The following abbreviations are used in this manuscript:

AuNPs	Gold Nanoparticles
AuNRs	Gold Nanorods
CPPs	Cell-Penetrating Peptides
CTAB	Cetyltrimethylammonium Bromide
DDS	Drug Delivery System
DLS	Dynamic Light Scattering
DSBs	Double-Strand Breaks
INFN	Istituto Nazionale di Fisica Nucleare
LEM	Local Effect Model
LSPR	Localized Surface Plasmon Resonance
MKM	Microdosimetric Kinetic Model
MRS	Magnetic Resonance Spectroscopy
NEXAFS	Near-Edge X-ray Absorption Fine Structure
SEGNAR	Synergic Effect of Gold Nanorods And Radiopharmaceutical
SF	Survival Fraction
SPECT	Single-Photon Emission Computed Tomography
SR	Synchrotron Radiation
TEM	Transmission Electron Microscopy
XAS	X-ray Absorption Spectroscopy
XPS	X-ray Photoelectron Spectroscopy
